# Supplementary figures and images for: Tandem-repeat protein domains across the tree of life
Source: PeerJ. 2015 Jan 13;3:e732. doi: 10.7717/peerj.732 (PMC4304861; doi:10.7717/peerj.732)

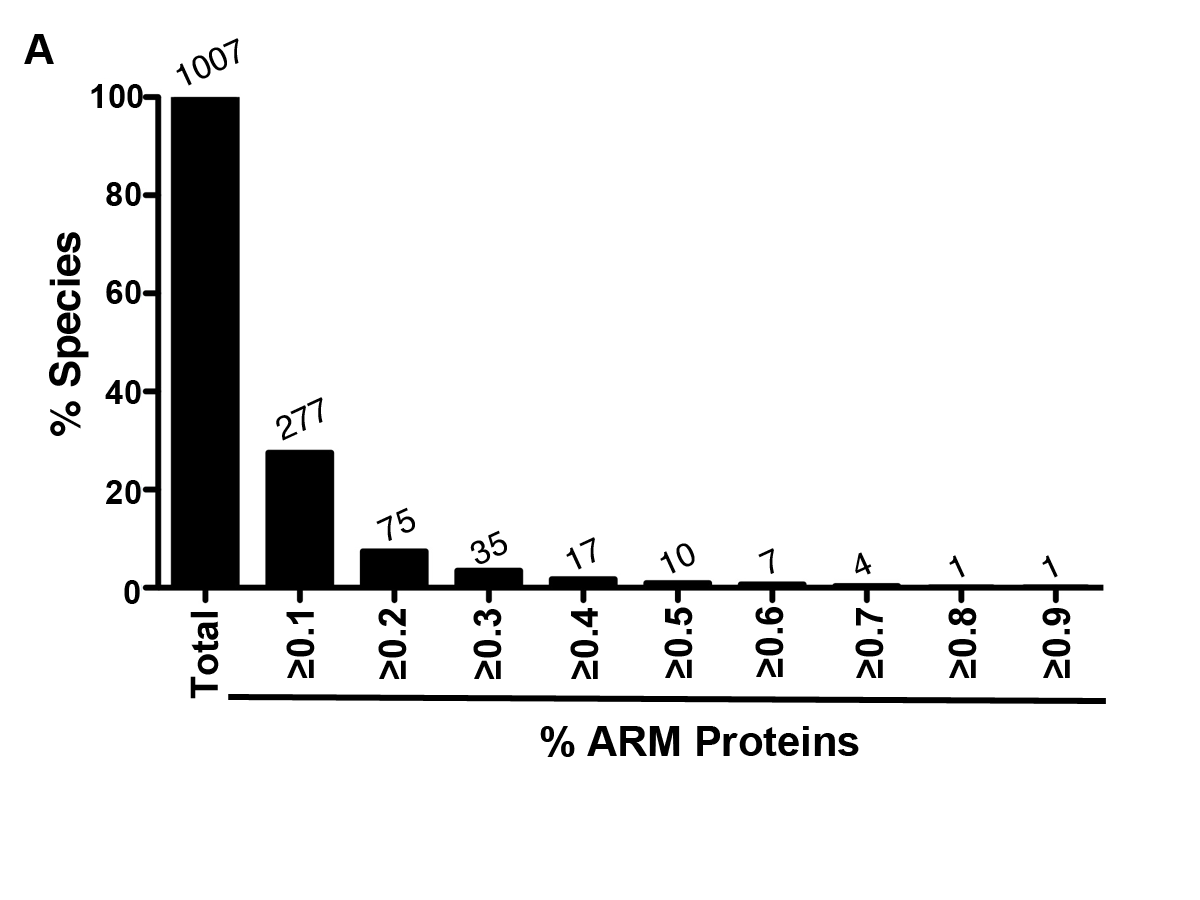

Supplement: Figure S1 — Bar graph of the percent of bacterial species analyzed (y axis) with the specified percent of ARM-containing proteins (x axis). The number above the bars on the graph lists the number of species with the specified percent of ARM-containing proteins. [file peerj-03-732-s001.png]

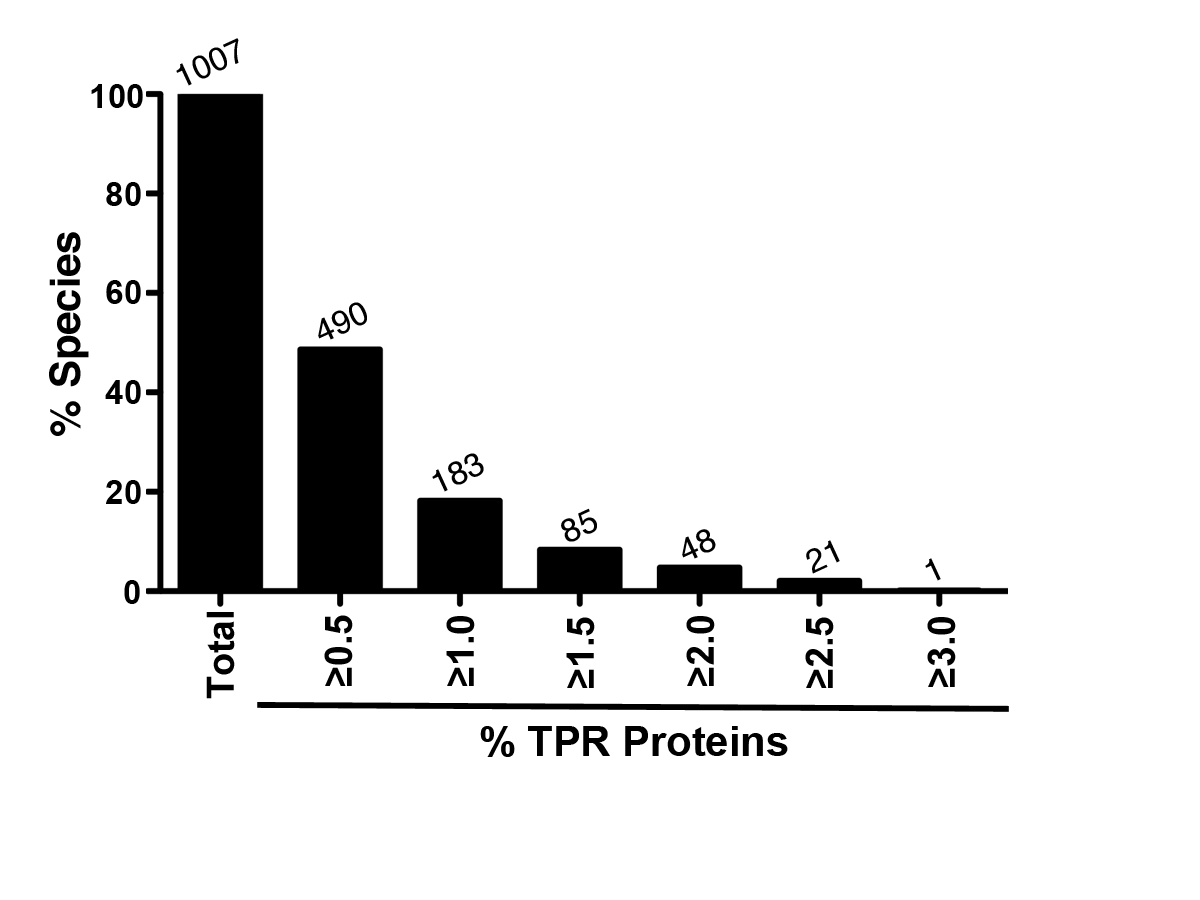

Supplement: Figure S2 — Bar graph of the percent of bacterial species analyzed (y axis) with the specified percent of TRP-containing proteins (x axis). The number above the bars on the graph lists the number of species with the specified percent of TPR-containing proteins. [file peerj-03-732-s002.png]

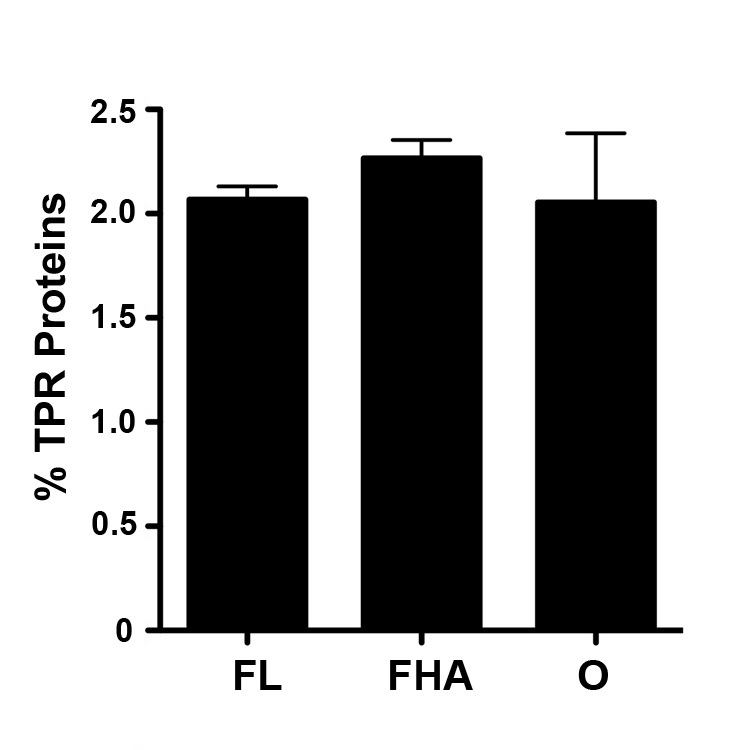

Supplement: Figure S3 — An average of the percent of TPR-containing proteins for all organisms of the same species was used for these analyses. Bar graph of the average percent of the proteome composed of TPR-containing proteins in species of free-living (FL), facultative host-associated (FHA) and obligate intracellular (O) bacteria with 1.5% of their proteome composed of TPR-containing proteins. Error bars represent standard error. (all comparisons, P > .05). [file peerj-03-732-s003.jpg]

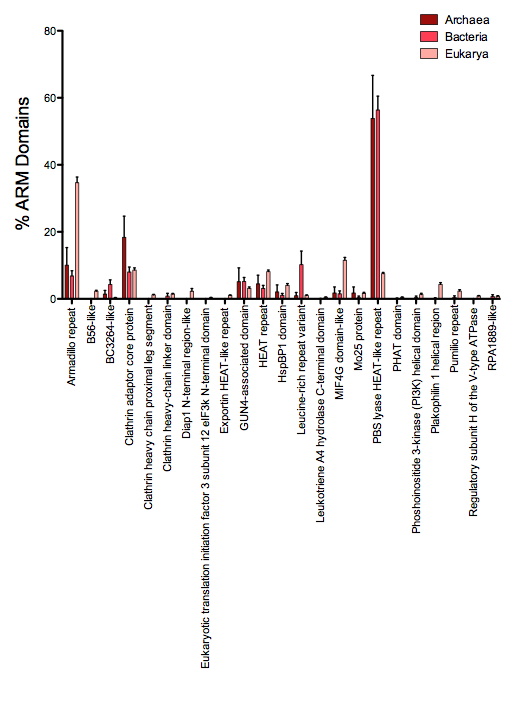

Supplement: Figure S4 — The ARM repeats of one bacterial and archaeal species within each phylogenetic family present in the data set (Tables S6 and S7), as well as 10 species of eukarya (each representing one phylogenetic family) were analyzed for their ARM domain composition. An average of the percent of ARM domains within each ARM family category was graphed. Error bars represent standard error. [file peerj-03-732-s004.png]
